# Supplementary material for: Antioxidant Systematic Alteration Was Responsible for Injuries Inflicted on the Marine Blue Mussel Mytilus edulis Following Strontium Exposure
Source: Antioxidants (Basel). 2024 Apr 14;13(4):464. doi: 10.3390/antiox13040464 (PMC11047646; doi:10.3390/antiox13040464)
Supplement: Supplementary file 1 [file antioxidants-13-00464-s001.zip › antioxidants-2921406-supplementary.pdf]

Supplementary Material

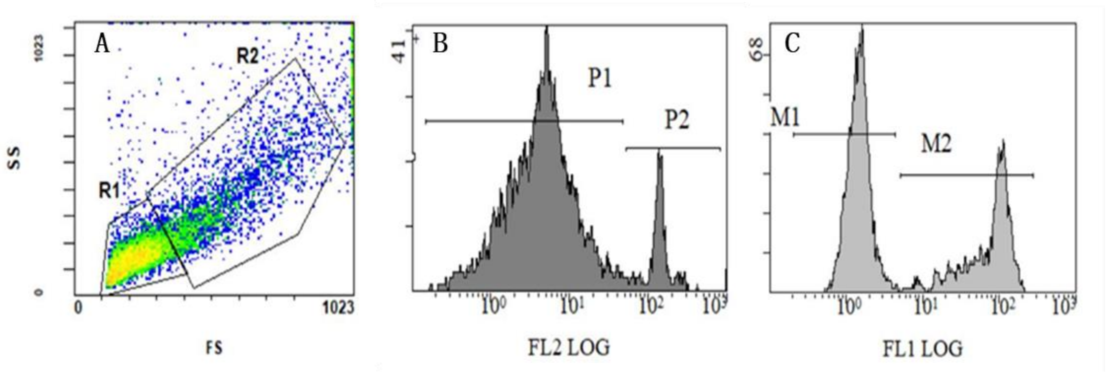

Figure S1 The classification of hemolymph in M. edulis by FCM. A: Cluster analysis of hemolymph; B: Mortality analysis of hemolymph, C: Phagocytic capability analysis of hemolymph.

Note: (A) R1: Hyalinocytes, R2: Granulocytes; (B) P1: the viability hemolymph, P2: the morality hemolymph; (C) M1: Hemolymph phagocytizing no beads; M2: Hemolymph phagocytizing one or more beads.
